# The contribution of *Coriandrum sativum* in enhancing *Oreochromis niloticus* rearing at sub-optimal temperatures: effect on growth, health status, survival rate, and resistance to *Aeromons Veronii*

**DOI:** 10.1186/s12917-023-03809-8

**Published:** 2023-11-30

**Authors:** Ahmed Abdou Said, Rasha M. Reda, Mohamed M. M. Metwally, Heba M. Abd El-Hady

**Affiliations:** 1 Department of Pharmacology, Faculty of Veterinary Medicine, Zagazig University, Zagazig, 44511 Egypt; 2 Department of Aquatic Animal Medicine, Faculty of Veterinary Medicine, Zagazig University, Zagazig,Sharkia, 44511 Egypt; 3 Department of Pathology, Faculty of Veterinary Medicine, Zagazig University, Zagazig, 44511 Egypt

**Keywords:** *Aeromonas veronii* antioxidant, *Coriandrum sativum*, *Oreochromis niloticus*, Suboptimal temperature

## Abstract

This study (60 days) was conducted to investigate the ability of diet enriched with *Coriandrum sativum* powder or its extract to protect *Oreochromis niloticus* health and survivability at suboptimal temperature (21 ℃). One hundred and twenty (33.14 ± 0.5 g) were divided into four groups; each group has three replicates.. The first control group fed on a basal diet. Second and third groups fed on diet enriched with 30 mg/kg coriander seed powder (CP) and coriander seed ethanolic extract (CE), respectively. The fourth group (OT) fed on diet enriched with 500 mg oxytetracycline/kg diet. The results revealed that CE exhibited a considerable improvement in hematological parameters, hepatic-renal functions, antioxidant status, and immunological markers as well as remarkably increased resistance against *Aeromonas veronii*. It could be concluded that feeding tilapia CE enriched diet at 30 mg/kg is a recommended strategy to enhance tilapia health and resistance to *A. veronii* infection reared at 21 ℃.

## Introduction

Aquaculture makes a considerable contribution to food security, particularly in developing countries [1, 2]. However, this industry has numerous challenges that must be overcome to ensure sustainable aquatic production in the future. Nile tilapia is mainly located in the tropics and subtropics, so low winter temperatures are considered a challenge for the high productivity of tilapia species. The cold months of the year and water temperature fluctuation between the seasons have a negative effect on fish feed intake, metabolism, and growth performance [3], immunity [4], physiological function [5, 6], and antioxidant activity [7]. Additionally, the relationship between fluctuations in water temperature and the incidence of bacterial infection, especially Aeromonas spp., has been recorded in previous studies [8–10].

*Aeromonus veronii* is one of nearly 31 species in the genus Aeromonas [11], and recent studies revealed that it is one of the reasons for economic losses and high mortalities in tilapia fish farms [12–14], as well as many other fish species [15–17].

Antibiotics are a potent treatment for infectious diseases; in addition, they can be used in a preventative medicated diet to lower mortality and morbidity rates while also promoting growth in farm rearing systems [18]. But unfortunately, antibiotic misuse, particularly in the aquatic ecosystem, has had a number of adverse effects on aquatic organisms, the environment, and public health [19]. The most harmful effects of antibiotic abuse include antibiotic accumulation in the environment over a long period of time, which disturbs the balance of microorganisms and results in the development of antibiotic-resistant bacteria and genes, as well as antibiotic accumulation in various fish tissues [19, 20]. As a result, it has recently become an urgent necessity to find alternatives that boost fish immune efficiency, disease resistance, and are environmentally friendly.

Medicinal plants are considered potential alternatives to antibiotics for preventing fish diseases; not only that, but they can also be used as feed additives, immunostimulants, and alternatives to some expensive imported feed components to reduce the cost of feeding fish [21–25]. A member of the Apiaceae family, coriander (*Coriandrum sativum*) is an aromatic and therapeutic plant [26]. Coriander seeds have vital components that preserve their pharmacological properties, such as linalool, terpenoids, linoleic acid, vitamin C, and minerals. It is also distinguished by several biological properties, including analgesic, antibacterial, antifungal, and anti-parasitic [26–28]. Recent studies have shown that a coriander-enriched diet has an improving effect on fish growth performance and protects them from a variety of pathogenic bacteria [29–31]. Furthermore, coriander-enriched diets therapeutic effectiveness against *A. veronii* was also proven [32]. As well, it acts as an antidote to some heavy metal toxicity [33, 34]. However, according to our information, the effect of a coriander-enriched diet on the growth performance and immune status of tilapia fish at suboptimal temperatures has not been studied.

Therefore, the goal of the current study was to determine the effects of a coriander seed powder or extract-enriched diet on the growth performance, hepatic-renal functions, antioxidant-immune response, and *Aeromonas veronii* resistance in *Oreochromis niloticus* at suboptimal temperature.

## Materials and methods

### Collection and preparation of coriander (*Coriandrum sativum*)

The coriander seeds gathered for this study were from a local market in Zagazig, Egypt. Before drying for 15 days at 29 ± 2 °C in the shade, the seeds had a thorough water wash. A pestle and mortar were used to grind the dried seeds into a fine powder. A portion of this fine powder was kept at 4 °C in a capped sterile bottle and utilized as a powder (CP). Another fraction of the fine powder was utilized for extraction to produce an ethanolic coriander extract (CE) according to the method reported by Ahmed, Reda [34]. The bioactive substances present in the C. sativum extract were identified using GC–MS analysis (Agilent Technologies, the Central Laboratories Network, National Research Centre, Cairo, Egypt). The findings showed that L-LINALOOL was the primary bioactive molecule, with the highest peak area% (92.52) at 10.107 retention time (RT, min.).

### Diet preparation and experimental protocol

According to the National Research Council's recommendations, four experimental diets (Table 1) were formulated to satisfy the nutritional requirements of tilapia fish [35]. The first diet (D1) acted as the basal control diet with no supplements. *C. sativum* powder (D2) and extract (D3), each at 30 mg/kg diet, were added to the second and third diets as supplements based on the results of a prior study on Nile tilapia by Ahmed, Reda [34]. The fourth (D4) was an antibiotic-supplemented meal that included oxytetracycline at a dosage of 500 mg/kg (Pharma Sweed, Egypt) [36]. One hundred and twenty *O. niloticus* (33.14 ± 0.5 g), all of which were apparently healthy, were purchased from a private fish farm in El-Abbassa, Sharkia, Egypt. *O. niloticus* were randomly distributed to four groups of triplicates (10 fish/replica, 30 fish/group), which were acclimated for 2 weeks before the beginning of the experiment. At the beginning of the first week of acclimation, the water's temperature was set to 27- 28 °C. Then the water temperature was gradually lowered for the fish during the second week of acclimatization by one degree each day, reaching 21 °C at the start of the feeding trial [37].

**Table 1:** Composition of experimental diets (g/kg)

Diet ingredients (%)	Diets			
	Control diet* (D1)	**Diet 2** **(D2)**	**Diet 3** **(D3)**	**Diet 4** **(D4)**
Fish meal (65.4% CP)	400	400	400	400
Soybean meal (44%)	200	200	200	200
Yellow corn	130	130	130	130
Wheat flour	150	150	150	150
Wheat Bran	20	20	20	20
Fish oil	70	70	70	70
Monocalcium phosphate	20	20	20	20
^(1)^ Vitamin mixture	4.5	4.5	4.5	4.5
^(2)^ Mineral mixture	5.5	5.5	5.5	5.5
*Coriandrum sativum* seed powder	-	0.03	-	-
*Coriandrum sativum* seed extract	-	-	0.03	-
Oxytetracycline	-	-	-	0.5
**Calculated compostion (% DM)**				
Crud Protein	38.90	38.90	38.90	38.90
Crude fat	10.50	10.50	10.50	10.50
Ash	5.84	5.84	5.84	5.84

^(1)^Vitamin mix (IU or mg kg diet): vitamin A, 16,000 IU; vitamin D, 8000 IU; vitamin K, 14.72; thiamin, 17.8; riboflavin, 48; pyridoxine, 29.52; cynocobalamine, 0.24, tocopherols acetate, 160; ascorbic acid (35%), 800; niacinamide, 79.2; calcium-D- pantothenate,73.6; folic acid, 6.4; biotin, 0.64 L-carnitine, 100

^(2)^Mineral mix (mg kg diet): Cu (CuSO4), 2.0; Zn (ZnSO4), 34.4; Mn (MnSO4), 6.2; Fe (FeSO4), 21.1; I (Ca (IO3)2), 1.63; Se (Na2SeO3), 0.18; Co (CoCl2), 0.24; Mg (MgSO4.H2O), 52.7

^*^Control normal diet [35]

The first control group (CONT) fed on a basal diet (D1). While the 2^nd^ (CP), 3^rd^ (CE), and 4^th^ (OT) groups fed on D2, D3, and D4, respectively for 60 days. The fish in each group were fed 3% biomass through three regular feedings during the day. The average dissolved oxygen (D.O.) concentration was 5 ± 1 mg/L, the pH level was 6.5 ± 0.5, the nitrite concentration was 0.05 mg/L, the nitrate concentration was 9 mg/L, and the ammonium concentration was 0.4 mg/L, which were maintained all over the experimental period. Throughout the trial period, about 25% of the water was replaced daily to maintain its quality.

### Growth indices evaluation

To evaluate growth performance, the fish were weighed every two weeks. The final body weight (FBW, g), weight gain (WG, g), daily weight gain (DWG, g), specific growth rate (SGR), and food conversion ratio (FCR) were evaluated [38].

### Blood samples collection

At the end of the trial, the fish were anaesthetized using a 100-mg/L benzocaine solution from Al-Nasr Pharmaceutical Chemicals Company. The blood samples were taken from the caudal vessels and placed in clean, sterilized tubes with ethylenediaminetetraacetic acid (EDTA) as an anticoagulant to determine the hematological marker. Additional blood samples were taken and centrifuged for 15 min at 3000 rpm to separate the serum and determine biochemical, antioxidant, and immunological markers.

### Hematological markers evaluation

The primary hematologic indices were determined manually using the method given by Groff and Zinkl [39]. A hemocytometer with Natt-dye Herrick's was used to measure the total red blood cell count (RBCs 10^12^/L). The hematocrit value (Hct; %) of the blood was determined by centrifuging it at 10.000 × g for 5 min. To measure hemoglobin concentration (Hgb; g/dl), the cyanmethemoglobin technique was applied. The following established formulas were utilized to determine mean corpuscular hemoglobin volume (MCV; fl), mean cell hemoglobin (MCH; pg), and mean corpuscular hemoglobin concentration (MCHC; g/dl):

$$\begin{array}{l}\mathrm{MCV\ }(\mathrm{fL})= [\mathrm{Hct }(\mathrm{\%})\mathrm{ \times }10]\ /\mathrm{\ Total\ RBCs}\\ \mathrm{MCH\ }(\mathrm{pg}) = [\mathrm{Hgb }(\mathrm{g}/\mathrm{dL})\mathrm{ \times }10]\ /\mathrm{\ Total\ RBCs}\\ \mathrm{MCHC\ }(\mathrm{g}/\mathrm{dL}) = [\mathrm{Hgb }(\mathrm{g}/\mathrm{dL})\mathrm{ \times }100]\ /\mathrm{\ Hct\ }(\mathrm{\%})\end{array}$$

Additional blood samples were obtained and transferred for centrifugation at 3500 rpm for 15 min to obtain blood plasma for the measurement of total protein (TP) [40], albumin (Al) [41], and globulin (Gl) by subtraction of serum albumin from total serum protein. The manual technique described by Dacie and Lewis [42] was used to determine platelets, total leukocytes (WBCs), and differential counts.

### Biochemical markers evaluation

Alanine aminotransferase (ALT), aspartate aminotransferase (AST), alkaline phosphatase (ALP), Urea, and creatinine levels were evaluated using ELISA (enzyme-linked immunosorbent assay, Thermo Fisher Scientific Inc., Egypt) according to commercial kits (MyBiosource Inc., USA) specific instructions [43].

### Antioxidant makers evaluation

The blood levels of total antioxidant capacity (TAC, ng/ml), superoxide dismutase (SOD, U/ml), and catalase (CAT, U/ml) were determined in accordance with the manufacturer's instructions using commercial enzyme-linked immunosorbent assay (ELISA) test kits from Cusabio Biotech Co., Ltd.

### Non-specific immune markers evaluation

The fish Nitric Oxide Synthase 2 Inducible ELISA Kit (MyBiosource Inc., USA) was used for measuring serum nitric oxide activity (NO, µmol/L) according to the manufacture instructions. According to Ellis [44], serum lysozyme activity was determined based on the lysis of *Micrococcus lysodeikticus* (Sigma Chemical Co.).

### Histopathological marker evaluation

The liver and head kidney of ten fish per group were collected and immediately fixed in a 10% neutral buffered formalin solution for 48 h. After fixation, the specimens were routinely processed for paraffin technique, and 4 µm thick tissue hepatic and renal tissue sections were prepared, stained with hematoxylin and eosin dyes following the protocol described by Suvarna, Layton [45], and investigated microscopically, recording any histological alterations.

### Challenge test

The *A. veronii* (131TF-ID) used in this investigation was isolated from tilapia fries that were naturally infected in Idku, Beheira Governorate, Egypt. Through rRNA gene sequencing, the isolate was identified as *A. veronii*, and it was recorded in the GenBank database (accession number: MN967136) [14]. The LD50 of A. veronii was previously determined to be 4.3 × 106 CFU/mL [14]. The bacterial stock was kept at -80 °C in tryptic soya broth with 15% glycerol until it was required.

Five fish per replicate were intraperitoneally injected with pathogenic *Aeromonas veronii* at dosages of 0.2 ml (4.3 × 10^6^, adjusted using McFarland standard tubes) following 60 days of feeding. The challenged fish were monitored for 7 days to record the clinical symptoms, postmortem lesions, and daily mortalities. To reisolate the bacterial strains, samples were obtained from the kidneys and liver of dead and clinically infected fish.

Using the mortalities of all replicates, the relative percentage survival (RPS) was calculated using the formula below [46]:

$$\mathrm{RPS }= 100 - [(\mathrm{treatment\ mortality }\div \mathrm{ control\ mortality})\mathrm{ \times\ }100]$$

### Animal ethics

The Institutional Animal Care and Use Committee of Zagazig University in Egypt authorized all experimental techniques, and all appropriate institutional guidelines were followed when caring for and utilizing animals in this work (ZU-IACUC/2/F/441/2022).

Fish were euthanized at the end of the experiment using an overdose of buffered benzocaine solution (> 250 mg/L for 10 min), then pithing fish to ensure euthanasia in accordance with the methods of euthanasia listed in the report of the American Veterinary Medical Association.

### Statistical analysis

Using IBM® SPSS® Statistical Package for Social Science for Windows (WINSPSS) version 25, the ANOVA one-way followed by Duncan's test at < 0.05 was used to determine the significance of the difference between the tested groups (Chicago, USA). The data were examined for normality using the Shapiro–Wilk W test and homogeneity of variances. The results are shown as the mean and standard error (M ± SE).

## Results

### Growth performances and survival rate

Table 2 presents the growth performance results. During the feeding trial (60 days). Supplementation with *C. sativum* extract dramatically enhanced growth indices and exhibited considerably greater FBW, WG, DWG, and SGR when compared to the CONT. FCR was considerably enhanced in CE and CP than in the CONT. *C. sativum* extract significantly improved the survival rate of fish (96.66%), while the fish fed the control diet showed the lowest survival rate (63.33%).

**Table 2:** Growth performance of *Oreochromis niloticus* fed on *Coriandrum sativum* seed powder or its extract-enriched diets for 60 days at suboptimal temperature

Parameters	CONT	CP	CE	OT	*P*-value
Initial body weight (g)	33.25 ± 0.15	33.06 ± 0.03	33.10 ± 0.13	33.16 ± 0.04	0.633
Final body weight (g)	50.99 ± 0.19^d^	62.12 ± 0.88^b^	67.20 ± 1.12^a^	55.16 ± 0.57^c^	0.000
Weight gain (g)	17.74 ± 0.35^d^	29.06 ± 0.85^b^	34.10 ± 1.26^a^	21.99 ± 0.62^c^	0.000
Daily weight gain (g)	0.25 ± 0.005^d^	0.41 ± 0.01^b^	0.48 ± 0.01^a^	0.31 ± 0.008^c^	0.000
Feed conversion ratio	3.32 ± 0.12^a^	2.16 ± 0.01^c^	1.93 ± 0.15^c^	2.68 ± 0.05^b^	0.000
Specific growth rate	0.71 ± 0.01^d^	1.05 ± 0.02^b^	1.17 ± 0.03^a^	0.84 ± 0.01^c^	0.000
Survival rate	63.33 ± 3.33^b^	76.66 ± 3.33^b^	96.66 ± 3.33^a^	73.33 ± 6.66^b^	0.004

Values with different superscripts [a, b, c, d] within rows are significantly different (*P* < 0.05)

CONT (control group): Fish fed with normal diet for 60 days at suboptimal temperature

CP: Fish fed with diet supplemented with *Coriandrum sativum* seed powder (CP) at 30 mg/kg for 60 days at suboptimal temperature

CE: Fish fed with diet supplemented with *Coriandrum sativum* seed extract (CE) at 30 mg/kg for 60 days at suboptimal temperature

OT: Fish fed with diet supplemented with 500 mg oxytetracycline/kg diet for 60 days at suboptimal temperature

### Hematological indices

The hematological index results are presented in Table 3. There were no significant differences seen in MCV, MCH, MCHC, neutrophils, eosinophils, basophils, and monocytes between the experimental groups. Only a significant decrease was recorded in RBCs, Hgb, platelets, WBCs, and lymphocytes in the OT-treated group in comparison to the CONT group, while there were no significant differences between CONT, CP, and CE. Total protein and albumin levels showed a significant decrease in CP compared with CONT. However, the globulin level showed a significant increase in CE in comparison with the CONT.

**Table 3:** Erythrogram and Leukogram of *Oreochromis niloticus* fed on *Coriandrum sativum* seed powder or its extract-enriched diets for 60 days at suboptimal temperature

Parameters	CONT	CP	CE	OT	*P*-value
**Erythrogram**					
RBCs (10^12^/l)	1.40 ± 0.26^a^	1.15 ± 0.09^ab^	1.44 ± 0.02^a^	0.91 ± 0.03^b^	0.092
Hct (%)	14.16 ± 1.74^ab^	12.87 ± 0.73^ab^	15.79 ± 2.38^a^	9.70 ± 0.55^b^	0.110
Hgb (g/dl)	4.90 ± 0.34^a^	4.73 ± 0.16^ab^	6.06 ± 0.82^ab^	4.26 ± 0.16^b^	0.112
MCV (fl)	111.75 ± 32.76	113.75 ± 13.17	109.79 ± 18.00	107.29 ± 9.62	0.996
MCH (pg)	39.92 ± 5.57	41.94 ± 7.68	42.01 ± 5.87	46.01 ± 0.15	0.550
MCHC (g/dl)	36.11 ± 6.33	37.05 ± 2.80	39.25 ± 4.37	44.43 ± 4.11	0.601
Total protein (g/dl)	6.83 ± 0.17^ab^	5.90 ± 0.05^c^	7.20 ± 0.15^a^	6.56 ± 0.08^b^	0.001
Albumin (g/dl)	4.00 ± 0.05^ab^	2.9 ± 0.05^c^	3.60 ± 0.05^b^	4.19 ± 0.25^a^	0.001
Globulin (g/dl)	2.83 ± 0.21^bc^	3.00 ± 0.11^b^	3.60 ± 0.10^a^	2.37 ± 0.20^c^	0.006
Platelets (× 10^3^/ mm^3^)	51.55 ± 9.27^a^	57.00 ± 9.81^a^	63.66 ± 9.93^a^	17.33 ± 3.17^b^	0.020
**Leukogram**					
WBCs (× 10^3^/mm^3^)	56.00 ± 0.17^a^	53.09 ± 5.53^a^	57.19 ± 2.10^a^	40.93 ± 1.74^b^	0.021
Lymphocyte (× 10^3^/mm^3^)	41.84 ± 3.98^a^	39.88 ± 5.56^ab^	45.22 ± 2.45^a^	28.77 ± 1.89^b^	0.066
Neutrophil (× 10^3^/mm^3^)	4.72 ± 1.57	2.93 ± 0.24	4.52 ± 1.32	4.70 ± 0.77	0.627
Eosinophil ((× 10^3^/mm^3^)	1.66 ± 0.66	1.21 ± 0.19	1.71 ± 0.63	1.28 ± 0.32	0.844
Basophil (× 10^3^/mm^3^)	0.07 ± 0.01	0.09 ± 0.04	0.06 ± 0.01	0.07 ± 0.01	0.864
Monocyte (× 10^3^/mm^3^)	7.70 ± 2.33	8.97 ± 0.54	5.64 ± 0.46	6.11 ± 1.37	0.375

Values are presented as the mean ± SE for three samples/replicate (*n* = 9/group). Values with different superscripts [a, b, c] within rows are significantly different (*P* < 0.05)

CONT (control group): Fish fed with normal diet for 60 days at suboptimal temperature

CP: Fish fed with diet supplemented with *Coriandrum sativum* seed powder (CP) at 30 mg/kg for 60 days at suboptimal temperature

CE: Fish fed with diet supplemented with *Coriandrum sativum* seed extract (CE) at 30 mg/kg for 60 days at suboptimal temperature

OT: Fish fed with diet supplemented with 500 mg oxytetracycline/kg diet for 60 days at suboptimal temperature

*RBCs* Red blood cells, *Hct* The hematocrit, *Hgb* Hemoglobin, *MCV* Mean corpuscular volume, *MCHC* Mean corpuscular hemoglobin concentration, *WBCs* White blood cells

### Biochemical parameters

**Fig. 1:**
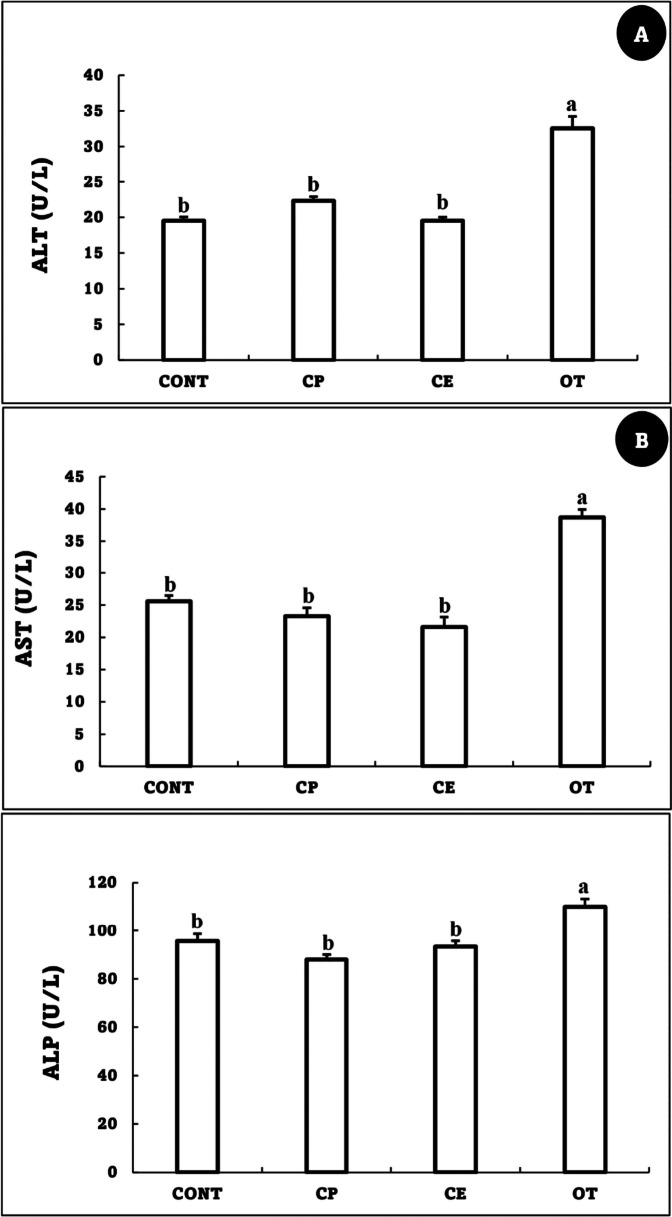
Alanine transaminase (ALT, U/L) (**A**), Aspartate transaminase (AST, U/L) (**B**) and alkaline phosphatase (ALP, U/L) (C) of *Oreochromis niloticus* fed on *Coriandrum sativum* seed powder or its extract-enriched diets for 60 days at suboptimal temperature. Values are presented as the mean ± SE for three samples/replicate (*n* = 9/group). The bars with different superscripts (a and b) are significantly different (*P* < 0.05, one-way ANOVA). CONT (control group): Fish fed with normal diet for 60 days at suboptimal temperature. CP: Fish fed with diet supplemented with *Coriandrum sativum* seed powder (CP) at 30 mg/kg for 60 days at suboptimal temperature. CE: Fish fed with diet supplemented with *Coriandrum sativum* seed extract (CE) at 30 mg/kg for 60 days at suboptimal temperature. OT: Fish fed with diet supplemented with 500 mg oxytetracycline/kg diet for 60 days at suboptimal temperature

**Fig. 2:**
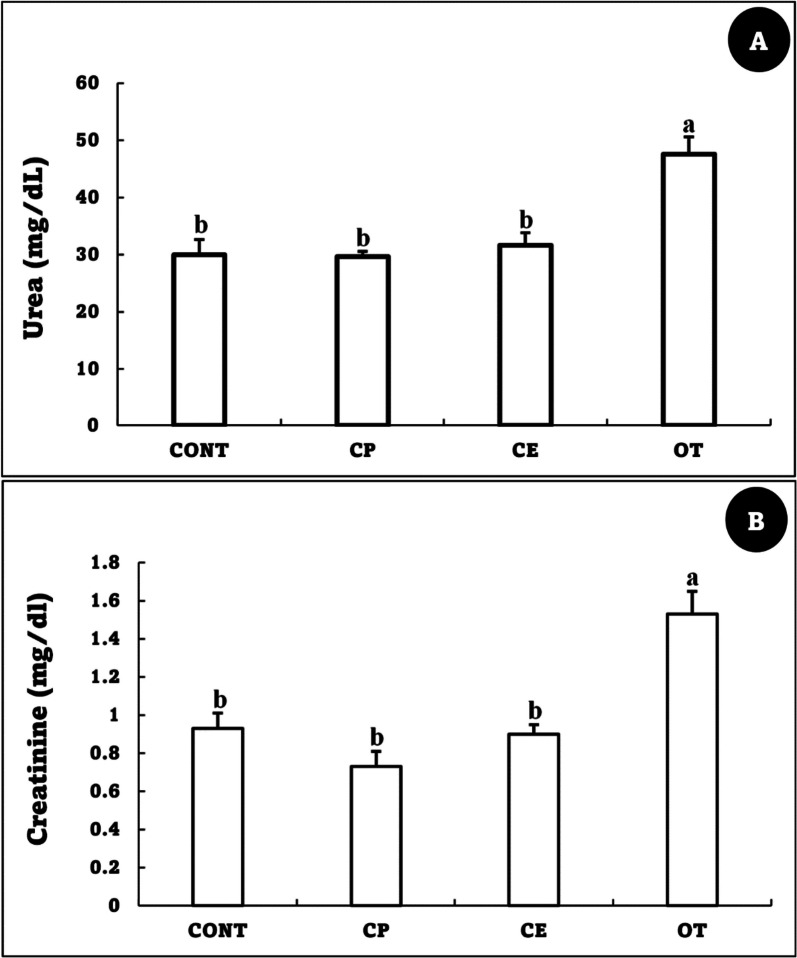
Urea (mg/dl) (**A**) and creatinine (mg/dl) (**B**) of *Oreochromis niloticus* fed on *Coriandrum sativum* seed powder or its extract-enriched diets for 60 days at suboptimal temperature. Values are presented as the mean ± SE for three samples/replicate (*n* = 9/group). The bars with different superscripts (a and b) are significantly different (*P* < 0.05, one-way ANOVA). CONT (control group): Fish fed with normal diet for 60 days at suboptimal temperature. CP: Fish fed with diet supplemented with *Coriandrum sativum* seed powder (CP) at 30 mg/kg for 60 days at suboptimal temperature. CE: Fish fed with diet supplemented with *Coriandrum sativum* seed extract (CE) at 30 mg/kg for 60 days at suboptimal temperature. OT: Fish fed with diet supplemented with 500 mg oxytetracycline/kg diet for 60 days at suboptimal temperature

While there were no significant changes between CONT, CP, and CE, there was only a significant increase in ALT, AST, ALP (Fig. 1), urea, and creatinine (Fig. 2) in the OT group in comparison to the CONT.

### Oxidant/antioxidant status

**Fig. 3:**
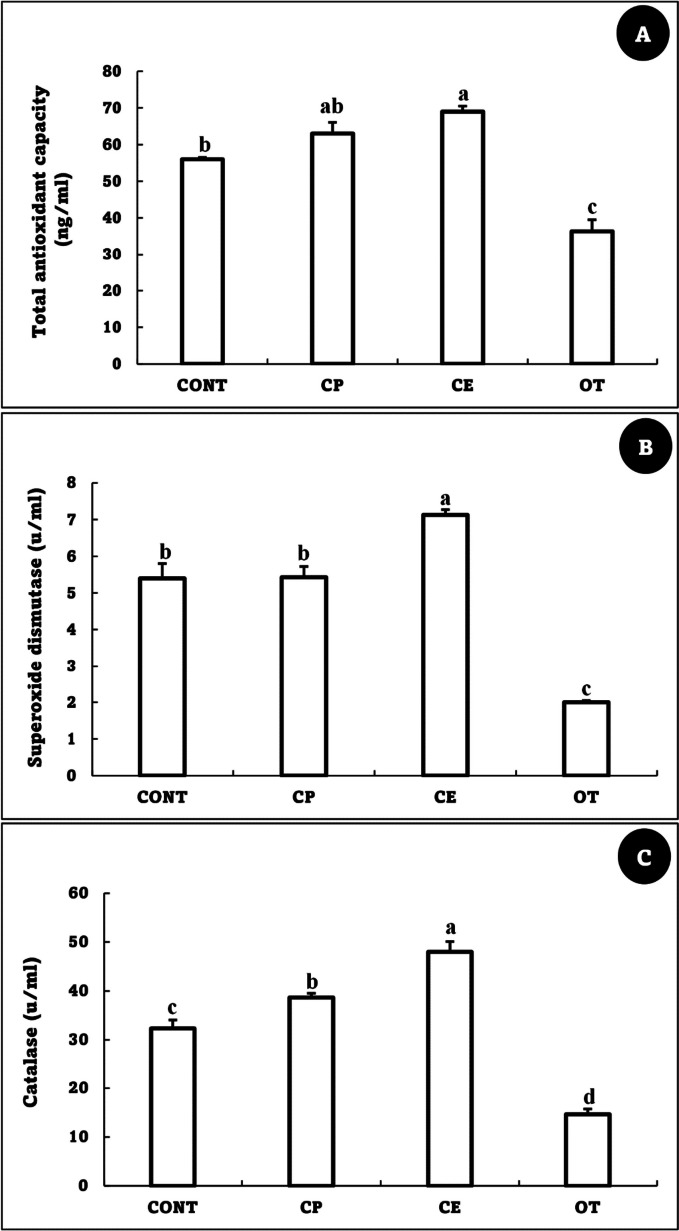
Total antioxidant capacity (ng/ml) (**A**), Superoxide dismutase (u/ml) (**B**), and Catalase (u/ml) (**C**) of *Oreochromis niloticus* fed on *Coriandrum sativum* seed powder or its extract-enriched diets for 60 days at suboptimal temperature. Values are presented as the mean ± SE for three samples/replicate (*n* = 9/group). The bars with different superscripts (a, b and c) are significantly different (*P* < 0.05, one-way ANOVA). CONT (control group): Fish fed with normal diet for 60 days at suboptimal temperature. CP: Fish fed with diet supplemented with *Coriandrum sativum* seed powder (CP) at 30 mg/kg for 60 days at suboptimal temperature. CE: Fish fed with diet supplemented with *Coriandrum sativum* seed extract (CE) at 30 mg/kg for 60 days at suboptimal temperature. OT: Fish fed with diet supplemented with 500 mg oxytetracycline/kg diet for 60 days at suboptimal temperature

The TAC indicated a substantial drop in the OT group when compared to the CONT group, while there were no appreciable changes between CONT, CP, and CE. On the other hand, SOD and catalase activity significantly decreased in the OT group compared to the CONT group; they dramatically increased in the CE group (Fig. 3).

### Immunological parameters

**Fig. 4:**
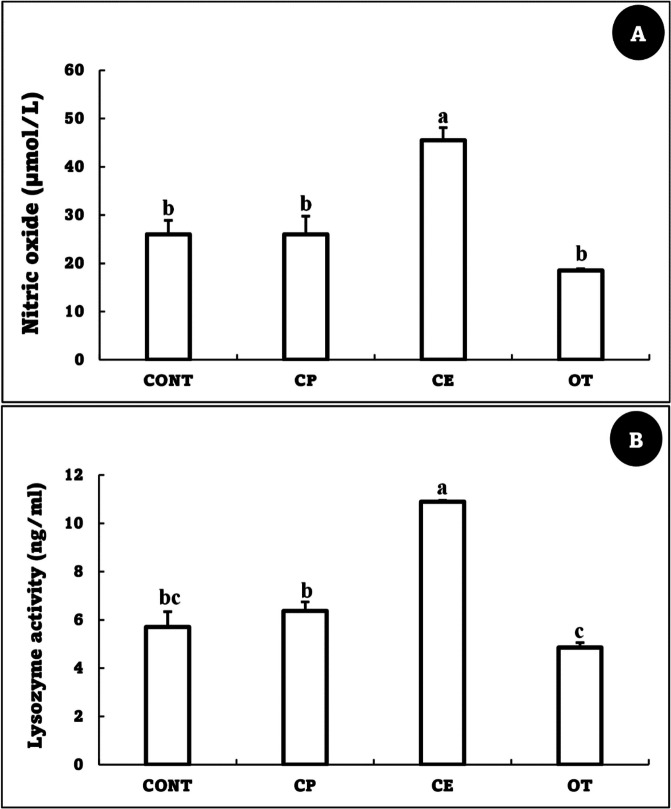
Nitric oxide (µmol/L) (**A**) and Lysozyme activity (ng/ml) (**B**) of *Oreochromis niloticus* fed on *Coriandrum sativum* seed powder or its extract-enriched diets for 60 days at suboptimal temperature. Values are presented as the mean ± SE for three samples/replicate (*n* = 9/group). The bars with different superscripts (a, b and c) are significantly different (*P* < 0.05, one-way ANOVA). CONT (control group): Fish fed with normal diet for 60 days at suboptimal temperature. CP: Fish fed with diet supplemented with *Coriandrum sativum* seed powder (CP) at 30 mg/kg for 60 days at suboptimal temperature. CE: Fish fed with diet supplemented with *Coriandrum sativum* seed extract (CE) at 30 mg/kg for 60 days at suboptimal temperature. OT: Fish fed with diet supplemented with 500 mg oxytetracycline/kg diet for 60 days at suboptimal temperature

The nitric oxide and lysozyme activities were markedly enhanced in the CE group than in CONT, whereas there were no notable differences between the CONT, CP, and OT groups (Fig. 4).

### Histopathological findings

**Fig. 5:**
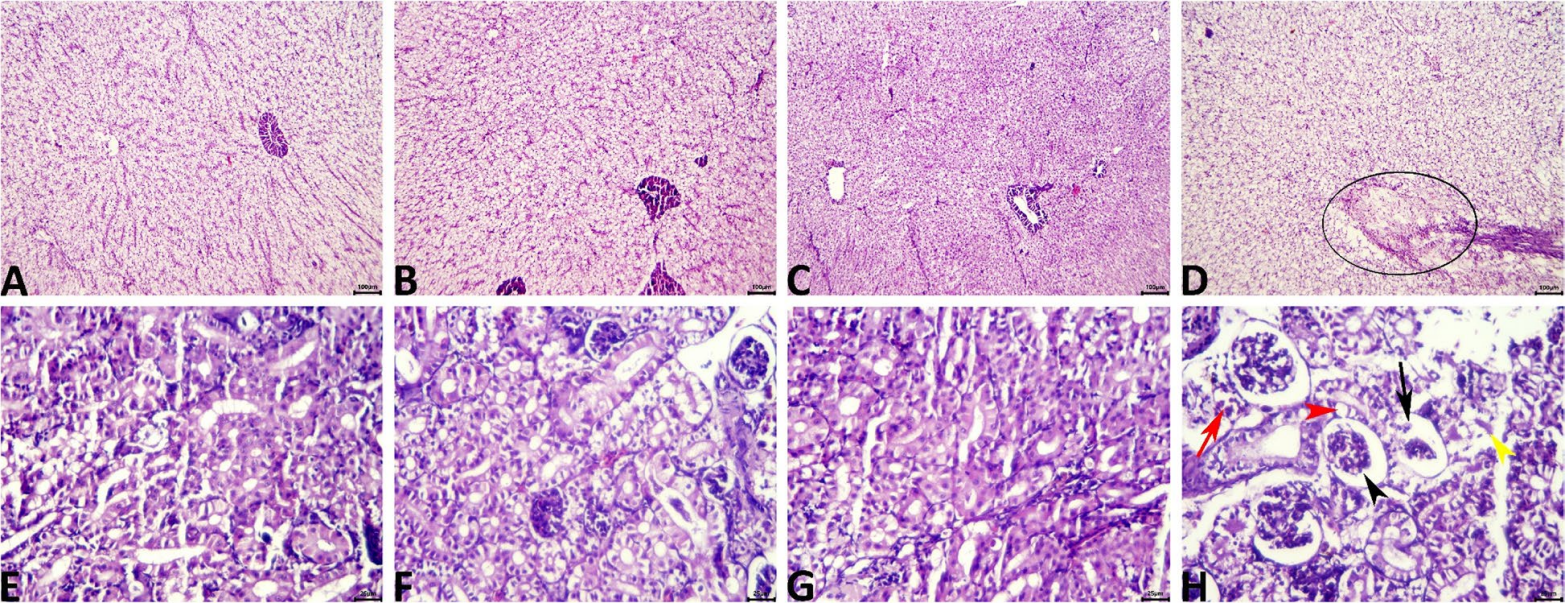
**A-D** Representative photomicrograph of H&E-stained (10x) hepatic tissue sections showing a normal histological picture in the control fish (**A**). A marked decrease in the lipoidal cytoplasmic vacuolations is seen in the hepatic tissue sections of the CP (**B**), and CE (**C**) groups. A focal lytic necrotic area containing numerous extravasated erythrocytes (ellipse) is seen in the OT (**D**) group. **E–H** Representative photomicrograph of H&E-stained (40x) renal tissue sections showing normal histological pictures in the control fish (**E**), CP (**F**), and CE (**G**) groups. Tubular vacuolation (red arrowhead), tubular necrosis (yellow arrowhead), glomerular atrophy (black arrowhead), glomerular necrosis (black arrow), and vascular congestion (red arrow) are seen in the OT (**H**) group

Normal histology was seen in all examined hepatic tissue sections of the CONT group (Fig. 5A), CP, and CE groups. Yet marked decrease in the lipoidal cytoplasmic vacuolations, associated with the presence of few numbers of lymphocytes particularly around the exocrine pancreas, besides, slight proliferations of the MMCs were observed in the hepatic tissue sections of the CP (Fig. 5B). These alterations were more pronounced in the CE group (Fig. 5C). Treatment with oxytetracycline induced variable degenerative, necrotic, and circulatory alterations in most specimens including increased cytoplasmic vacuolation, single-cell necrosis, and vascular congestion. Additionally, a few tissue sections of the OT group exhibited focal lytic necrosis infiltrated with numerous extravasated erythrocytes (Fig. 5D).

The kidneys of the CONT (Fig. 5E), CP (Fig. 5F), and CE (Fig. 5G) groups showed normal histological pictures without any variations between these groups. Controversy, most tissue sections of the OT fish manifested various degenerative changes such as tubular vacuolations and necrosis, cast formation, glomerular atrophy, and necrosis, and congestion of the interstitial vasculatures (Fig. 5H).

### *Aeromonas veronii* resistance

**Fig. 6:**
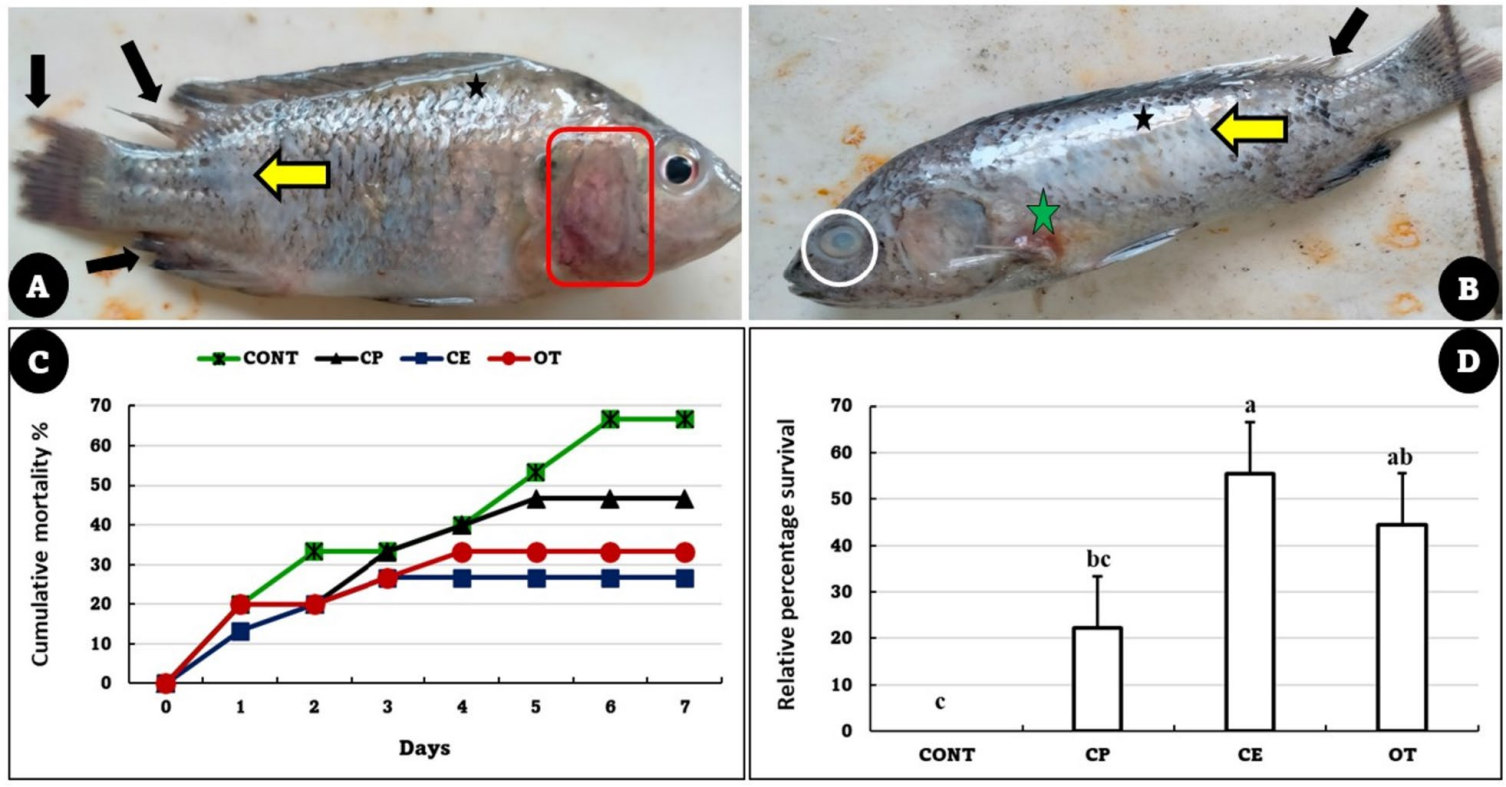
**A** and **B** Main symptom of fish from control group (Fish fed with normal diet for 60 days at suboptimal temperature then challenged with *Aeromonas veronii*) showing detached scales (yellow arrow), fin rot (black arrow), hemorrhage at the base of pectoral fin (green star), high mucus secretion (black star), eye turbidity (white circle), congestion of the operculum (red rectangle). **C** and **D** Cumulative mortality rate and Relative percentage survival of *Oreochromis niloticus* fed on *Coriandrum sativum* seed powder or its extract-enriched diets for 60 days at suboptimal temperature and challenged with pathogenic *Aeromonas veronii* for 7 days. CONT (control group): Fish fed with normal diet for 60 days at suboptimal temperature. CP: Fish fed with diet supplemented with *Coriandrum sativum* seed powder (CP) at 30 mg/kg for 60 days at suboptimal temperature. CE: Fish fed with diet supplemented with *Coriandrum sativum* seed extract (CE) at 30 mg/kg for 60 days at suboptimal temperature. OT: Fish fed with diet supplemented with 500 mg oxytetracycline/kg diet for 60 days at suboptimal temperature

After 60 days of the feeding trial, *O. niloticus* challenged with *A. veronii* showed detachment of the scales, destruction of fin rays with fin rot, hemorrhage at the base of fins, especially the pectoral fin, congestion of the gill cover with excessive mucus secretion, and eye turbidity (Fig. 6A and B). The cumulative mortality rate was elevated in the control group (66.66%), followed by the CP (46.66%), OT (33.33%), and CE (33.33%) groups (Fig. 6C). The highest RPS was recorded with the CE group, while there was no significant difference between the CP and OT groups (Fig. 6D).

## Discussion

The negative impact of suboptimal and cold temperatures on tilapia health has been shown in previous studies [4–6, 47]. The objective of the current study was to determine how *C. sativum* affected *O. niloticus* reared at suboptimal temperatures in terms of growth, health condition, survival rate, and resistance to *A. veronii*.

About 25–32 °C is the ideal temperature range for tilapia to grow and develop normally [48]. This study exhibited that CE was able to improve the growth performance and survival rate of tilapia fish raised at suboptimal temperatures compared to the CONT group. The results of Farsani, Hoseinifar [31] have shown the enhancement of *Oncorhynchus mykiss* growth performances when fed diets supplemented with CE at a rate of 2% for 8 weeks. Also, the dietary administration of 10–20 g coriander seed powder/kg for 150 days has improved the growth performance and survival rate of *Dicentrarchus labrax* [49]. The enhancement of the growth performance could be related to (i) the ability of coriander to reduce counts of pathogenic intestinal bacteria that compete for nutrients by their antibacterial effect [49, 50]. (ii) The antioxidant constituents in CE prevent intestinal mucosal damage and enhance absorption [51, 52]. (iii) The ability of CE to stimulate bile and digestive enzyme secretion, which improves the digestion process [50].

*O. niloticus* is extremely sensitive to any drop in the ambient temperature, which causes poor growth and massive mortality [53, 54]. The current study demonstrated an improvement in the survival rate of fish fed a diet supplemented with CE (96.66%) compared to those fed a basal diet (63.33%) during the experimental period under suboptimal temperature conditions. This suggests that CE may benefit tilapia under low-temperature stress. This finding could be related to the richness of CE with phenolic (especially linalool) and flavonoid compounds [29, 51, 55], which protect fish from the oxidative stress resulting from the suboptimal temperature. According to the data we have gathered, this study is considered the first to demonstrate the beneficial effect of coriander extract on the survival rate of tilapia reared under suboptimal temperature conditions.

In aquaculture, hematological studies are used to assess the health and welfare of fish. These analyses are thought to be a good indicator of a variety of environmental parameters, such as infections, diet, and water quality [56]. RBCs, Hgb, platelets, WBCs, and lymphocytes of the OT-treated group in the current study were significantly lower than those of the CONT group, while there was no significant difference between CONT and the other experimental group. According to earlier research, fish exposed to antibiotics may have a variety of hematological alterations, including an increase or decrease in leukocyte count and red blood cell parameters (RBC, Ht, and Hb). Similar to our results, Omoregie and Oyebanji [57] found that oxytetracycline-induced significant reductions in leukocyte, erythrocyte, thrombocyte, hematocrit, and hemoglobin values of *O. niloticus* after being fed on diets incorporating varying concentrations of the drug (5.00, 2.50, 1.25, and 0.63%) for 8 weeks indicated an anemic response. On the other hand, the globulin level indicated a substantial increase in the CE group as compared to the CONT group in the current study. Globulin is a group of serum proteins (e.g., enzymes, complement, and immunoglobulin) formed by liver and plasma cells [58]. Therefore, as reported by Ola and Sofolahan [59], the increase in globulin levels in the CE group may be attributable to the presence of linalool and phenolic compounds, which in turn improve liver functions and hematological markers.

The liver is primarily responsible for the metabolism of antibiotics, while the kidneys are responsible for their active form of elimination. The results of the current study showed that the renal and liver functions of the OT group were disturbed, as evidenced by elevated levels of urea, creatinine, AST, ALT, and other compounds. Notably, oxytetracycline induced similar disturbances in the liver and kidney function of *O. niloticus* at 500 mg/kg of diet for 14 days [60] and 100 mg/kg of diet for 84 days [61] and *O. mykiss* at 2.5 g/kg of diet for 14 days [62]. Other types of antibiotics have also been shown to have a similar effect on liver and kidney function. For example, gentamicin injection at a dose of 36 mg/kg caused significant disruption in the trunk kidney and liver of *O. niloticus* and raised ALT and AST levels [63]. On the other hand, the addition of coriander, whether as an extract or powder, did not negatively impact the liver or kidney activities in the present study. This may be due to active coriander seed ingredients like linalool, linoleic acid, vitamin C, terpenoids, and minerals, which maintain the physiological functions of the liver and kidneys [50, 64–66].

Exposure to suboptimal temperatures leads to the production of reactive oxygen species (ROS). Excessive ROS generation impairs physiological processes, damages DNA, induces cellular apoptosis, and suppresses the immune system [7, 37, 47]. In the current study, TAC, SOD, and catalase activity significantly decreased in the OT group compared to the CONT group. Previous studies have proven that OT overdose is linked to decreased tissue antioxidant indicators, damages mitochondria, impairs the respiratory chain, and peroxides the lipids in membranes [67–69]. The results are in line with previous reports regarding Limbu, Zhang [70], who recorded that hepatic SOD and GST activities decreased and MDA level and AST activity increased in *O. niloticus* fed on a diet supplemented with 80 mg/kg of oxytetracycline for 35 days, which indicates oxidative damage. On the other hand, the group that was fed a diet containing CE in the present study was distinguished by an increase in SOD and catalase activity due to the richness of CE in phenolic compounds, the most important of which is linalool [29, 71]. Strong antioxidant properties found in linalool enable it to scavenge ROS produced by a variety of stressors [72, 73]. Similar to our results, Das (2023) reported a significant increase in the serum SOD activity of *O. niloticus* in the coriander oil-treated groups compared to the control after 60 days. These findings clarify the reasons for the improvement of hematological and biochemical parameters in the CE group in the present study.

Fish immune systems are weakened by the suboptimal temperature, which also interferes with vital physiological and biochemical processes [4, 37]. In the current study, at the suboptimal temperature, the nitric oxide and lysozyme activities were markedly enhanced in the CE group compared to the CONT group. Different functional components, including phenolic compounds, notably linalool, flavonoids, and alkaloids, give *C. sativum* its pharmacological, immunostimulant, and antioxidant properties [50, 74]. Similarly, the respiratory burst, myeloperoxidase, lysozyme, and antiprotease activities of *O. niloticus* fed a diet supplemented with 1.5 and 2% coriander oil for 60 days exhibited significant increases compared to the control [29]. Ashry, Habiba [49] recorded a significant increase in the lysozyme and phagocytic activities of *Dicentrarchus labrax* fed 10 and 20 g of coriander seed powder per kg of diet for 150 days. Moreover, *O. mykiss* fed a diet enriched with 2% coriander seed extract for 8 weeks showed significant improvements in lysozyme and alternative complement activities [31].

Histopathology is a well-known and strong method for identifying changes in the normal condition of live tissues and potentially their causative agent that cannot be noticed by the naked eye [75]. In the present study, histopathological abnormalities were only observed in the liver and renal tissues of fish in the OT group. This confirms the earlier findings in this study regarding disturbances in hepatic and renal function, as well as antioxidant activity in the OT group. Abraham, Roy [76] observed histological changes in hepatic and renal tissues of *O. niloticus* treated with oxytetracycline at 80 mg/kg biomass/day that progressed from mild to moderate on day 10. These results are similar to those of Islam, Rasul [77] for *Barbonymus gonionotus*, Krupesha Sharma, Sumithra [78] for *Trachinotus blochii*, Solanki, Avunje [79] for *Cirrhinus mrigala*, and Rodrigues, Antunes [80] for *O. mykiss*, despite differences in the type of fish, the dose of oxytetracycline, and the duration of exposure. On the other hand, the recent study conducted by Yigit and Kocaayan [81] confirmed that no pathological findings were observed in any organ (gill, liver, or kidney) of O. mykiss in the coriander group at the different concentrations (20, 50, 70, 100, 150, 200, 300, 400, 500, and 600 mg L^−1^). Several studies also supported the idea that *C. sativum* protects liver and kidney cell tissues from oxidative damage brought on by various stressors [29, 82, 83].

Fish exposure to suboptimal temperatures has negative effects on their physiological functions and immune response, as well as impairing their ability to resist pathogens and thus compromising their overall health [4]. In the present study, CE enhanced tilapia resistance to *A. veronii* and increased RPS. This is in line with the results of Das, Pradhan [29], who recorded an increase in the survival rate of *O. niloticus* challenged with *A. hydrophila* and fed on diets supplemented with coriander oil in a dose-dependent manner compared to the control group. Similar to this, *O. mykiss* fed on a diet supplemented with CE at 2% and challenged with *Y. ruckeri* exhibited the lowest mortality rate (40%), compared to the control group (60%) [31]. Linalool and other bioactive components, which can destroy the bacterial cell membrane and result in cell death, may be responsible for the antibacterial effect of CE and the enhancement of survival rates [32, 84]. In addition, the foregoing results in the current study indicated an enhancement in the antioxidant capacity and immune status of fish fed on diets enriched with CE, so they were more resistant to *A. veronii* infection. These findings are supported by previous reports that have proven the immunostimulant effect of the CE to enhance the resistance of various types of fish against pathogens [29–31].

## Conclusion

These findings demonstrated that dietary supplementation with *C. sativum* extract at 30 mg/kg can lessen the immunosuppressive effects of suboptimal temperature in *O. niloticus* and increase resistance to *A. veronii* infection.

## Data Availability

All data generated or analyzed during this study are included in this published article.
